# Lycopene: Food Sources, Biological Activities, and Human Health Benefits

**DOI:** 10.1155/2021/2713511

**Published:** 2021-11-19

**Authors:** Usman Mir Khan, Mustafa Sevindik, Ali Zarrabi, Mohammad Nami, Betul Ozdemir, Dilara Nur Kaplan, Zeliha Selamoglu, Muzaffar Hasan, Manoj Kumar, Mohammed M. Alshehri, Javad Sharifi-Rad

**Affiliations:** ^1^National Institute of Food Science and Technology, University of Agriculture, Faisalabad, Pakistan; ^2^Bahçe Vocational High School, Osmaniye Korkut Ata University, 80500 Osmaniye, Turkey; ^3^Department of Biomedical Engineering, Faculty of Engineering and Natural Sciences, Istinye University, 34396 Sariyer, Istanbul, Turkey; ^4^Department of Neuroscience, School of Advanced Medical Sciences and Technologies, Shiraz University of Medical Sciences, Shiraz, Iran; ^5^Department of Cardiology, Faculty of Medicine, Nigde Ömer Halisdemir University, Nigde, Turkey; ^6^Department of Nutrition and Dietetics, Faculty of Health Sciences, Karabuk University, Karabuk 78050, Turkey; ^7^Department of Medical Biology, Faculty of Medicine, Nigde Ömer Halisdemir University, Nigde 51240, Turkey; ^8^Agro Produce Processing Division, ICAR-Central Institute of Agricultural Engineering, Bhopal 462038, India; ^9^Chemical and Biochemical Processing Division, ICAR-Central Institute for Research on Cotton Technology, Mumbai 400019, India; ^10^Pharmaceutical Care Department, Ministry of National Guard-Health Affairs, Riyadh, Saudi Arabia; ^11^Phytochemistry Research Center, Shahid Beheshti University of Medical Sciences, Tehran, Iran; ^12^Facultad de Medicina, Universidad del Azuay, Cuenca, Ecuador

## Abstract

As an antioxidant, lycopene has acquired importance as it prevents autoxidation of fats and related products. Tomatoes are an important agricultural product that is a great source of lycopene. It contains many vitamins and minerals, fiber, and carbohydrates and is associated with various positive effects on health. The antioxidant potential of tomatoes is substantially explained with lycopene compounds. Diet is a major risk factor for heart diseases which is shown as the most important cause of death in the world. It has been observed that the lycopene taken in the diet has positive effects in many stages of atherosclerosis. The serum lipid levels, endothelial dysfunction, inflammation, blood pressure, and antioxidative potential are mainly affected by lycopene. These natural antioxidants, which can also enhance the nutritional value of foods, may lead to new ways if used in food preservation. In this review study, the antioxidant potential and cardiovascular protection mechanism of lycopene are discussed.

## 1. Introduction

Natural antioxidants' importance in human health has increased by the scientific development of antioxidants recently. Many communities across the globe benefitted from natural antioxidant by treating various disorders and improving the human health. Unhealthy diet is considered as a major cause of many pathological conditions, including cardiovascular diseases. The key cause of cardiovascular diseases is the proinflammatory state, excessive production of reactive oxygen species, and irregular plasma lipid levels. There are many natural molecules that provide cardiovascular protection and prevent the risk of diseases. Antioxidants are among the most important molecules in the prevention of cardiovascular diseases by providing cardio protection. Plant and plant-based extracts are used for medicinal purposes such as prevention of diseases because the protection system in plants prevents various stress damages of active oxygen [1–3]. Plants repair their cells and genetic materials such as radical scavenging compounds (ascorbic acid, carotenoids, and synthesized components) by means of antioxidant enzymes (super oxide dismutase, catalase, and peroxidase). Inhibiting the oxidation of other molecules is the capability of antioxidants. These natural compounds are typically reducing agents such as polyphenols, thiols, carotenoids, and ascorbic acid. Consequently, most effective plants and herbs against oxidative stress have been researched for their importance on human health [4, 5].

**Figure 1:**
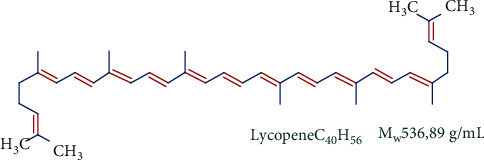
Structure of lycopene.

The chemical diversity that drives the pharmaceutical industry has been derived from natural products for centuries. Chemical existences resulting from the living creatures such as microorganisms, marine organisms, and plants are “natural products.” These compounds contribute towards their multitargeted activity and have outstanding drug-like properties and complex chemical diversity. These naturally occurring chemicals have been used for the prevention of pathological conditions due to their biological and therapeutic effects. Modern drug production from medicinal plants is a multifaceted approach that connects molecular, phytochemical, and biological techniques. Plant-based drug research offers new clues for many biological conditions [6, 7]. Natural antioxidants such as lycopene have gained importance in preventing oxidation of fats/oils and foods. Lycopene is a lipophilic carotenoid hydrocarbon pigment found in red, pink, and orange fruit and vegetables such as tomatoes, apricots, melons, papayas, grapes, peaches, watermelons, and cranberries (Figure 1). The lycopene extraction could serve as a food grade source of carotenoid [8, 9].

Lycopene has been associated with numerous biological activities. This review paper discusses deeply about its main biological effects and especially focuses on its health-promoting effects such as anticancer, antioxidant, cardioprotective, neuroprotective, improves sleeping behavior, anti-inflammatory, antiplatelet aggregative, and antihypertensive action. Moreover, its excessive consumption can lead to lycopenemia and additional supplementation apart from fruits and vegetable may lead to disturbance in pregnancy.

Considering the various health benefits of lycopene in human health, the current review presents various sources, side effects, and biological activities of lycopene.

## 2. Food Sources and Bioavailability

**Figure 2:**
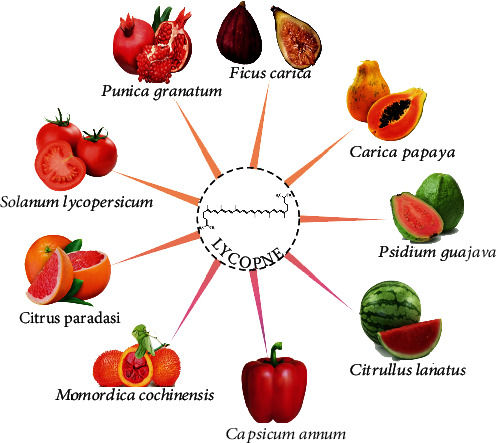
Lycopene sources [13].

**Figure 3:**
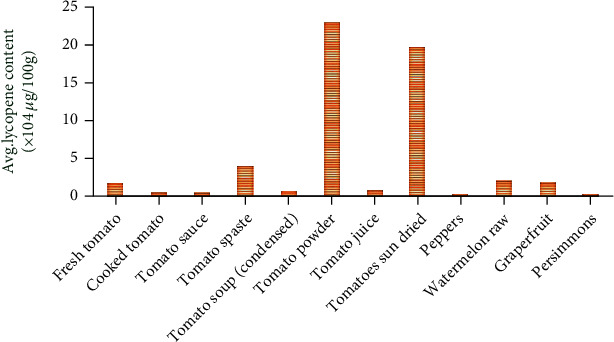
Lycopene content in food sources [13].

Many natural products contain lycopene. Red fruits and vegetables such as tomatoes, pink guavas, apricots, watermelons, and pink grapefruits are important sources of lycopene. Tomato is a major contributor of lycopene and is also a considerable source of vitamins K, A, C, fiber, and carbohydrates, besides some amounts of iron, potassium, phosphorus, and sulfur. In addition, it contains low sodium, fat, and calories. Various sources of lycopene are shown in Figure 2. Besides, some of the dietary sources of lycopene include processed tomato products like tomato paste, ketchup, sauce juices, and soups [10]. Lycopene bioavailability is profoundly affected by dietary content. Since it is a lipid-soluble substance, consumption with dietary sources of fat amplifies lycopene bioavailability [11]. There are studies showing that lycopene bioavailability in heat-treated tomatoes is increased compared to fresh tomatoes. In addition, it has been observed that tomatoes grown in the field contain higher levels of lycopene than tomatoes grown in the greenhouse [12]. The lycopene sources and concentration of lycopene in fresh and processed tomato products are shown in Figures 2 and 3.

## 3. Possible Side Effects

### 3.1. Lycopenemia

Lycopenemia is a clinical condition characterized by pigmentation of the skin color from yellow to orange. It is caused by the accumulation of lycopene depending on the excess consumption of lycopene resources taken through the diet. Lycopene deposits take place in the stratum corneum, which has high lipid content and affinity against lycopene. Often, the diagnosis of lycopenemia is determined by a history of diet. With the changes in diet, the symptoms are eliminated [14].

### 3.2. Lycopene in Pregnancy

The consumption of lycopene in foods taken through diet during pregnancy and lactation is generally safe. However, supplemented lycopenes taken in addition to food lycopene taken during pregnancy are probably not safe. In a study, 2 mg of lycopene was used daily as a supplement starting from 12 to 20 weeks of pregnancy until birth. As a result, the proportion of preterm and low birth weight infants increased [15]. However, in a different study using lycopene supplements, these problems were not seen. There is not enough information about the reliability of lycopene supplementation during breastfeeding. Consequently, it is recommended to avoid supplementing lycopene in addition to food-borne lycopene during pregnancy and lactation [16].

In a study conducted on pregnant women, lycopene and vitamin C levels were found to be significantly lower in preeclamptic pregnant women compared to healthy pregnant women. Also, there was an increase in oxidative stress markers (glutathione peroxidase, superoxide dismutase, and malondialdehyde). As a result, it was thought that additional dietary antioxidants (lycopene and vitamin C) might be beneficial in the prevention of preeclampsia [17].

## 4. Biological Activities of Lycopene

### 4.1. Antioxidant Effects

Tomatoes are considered as a substantial agricultural commodity globally. They consist of peel, locular matter, and pericarp. In the locular cavity, there are gel-like parenchyma cells around the seeds. In the current scenario, there has been an increase in consumers' preference for natural products due to the rising knowledge about the health disorders caused by synthetic additives. Some of these additives used in the food industry are flavoring agents, coloring agents, and diverse preservatives. Antioxidants act as free radical scavengers, and due to these properties, they prevent food components from reacting with oxygen and ultimately delaying spoilage of perishable items [18–22]. Lycopene can neutralize reactive species such as hydrogen peroxide, hydroxyradicals, and nitrogen dioxide. The red color of many red- or light red-colored fruits is due to the presence of lycopene and other carotenoids in them. As a natural pigment, lycopene is biosynthesized by plants and various microorganisms. Lycopene protects plants against photosensitization by absorbing light during photosynthesis. The colors of flowers and fruits are due to both the decrease in chlorophyll content as they mature and the lycopene and other carotenoids in their contents [23, 24].

As awareness about the possible negative effects of synthetic food antioxidants proliferates, the use of natural antioxidants as preservatives is increasing. This can also enhance the nutritional value of foods. As these antioxidants continue to be used in food preservation, they may contribute to the development of new ways to use natural antioxidants instead of synthetics [25–29].

The antioxidant potential of tomatoes and tomato products is attributed to the rich lycopene content, and lycopene extraction methods from tomatoes have been developed. In previous years, organic solvents such as benzene, hexane, ethyl ether, acetone, and ethyl acetate and different solvent combinations were used for lycopene extraction. However, these solvents used alone or in combination are not effective at maximum extraction because they cannot dissolve cellulose and pectin, which are the cell wall components [30].

Tomato, which works as a natural antioxidant, helps prevent bleeding and is protective against pulmonary diseases [31]. It acts as a blood purifier as well. Not only the whole tomato but also its products are a good source of lycopene. Tomato pulp and tomato paste are two important lycopene source products. Tomato paste is obtained by removing the peel and seeds from ripe tomatoes and cooking for a few hours. The resulting thick concentrate is tomato paste. Instead, tomato pulp is an unprocessed form. Fresh tomatoes are pressed through the dough machine, and then, the seeds and the shell are separated and finally tomato pulp is obtained [32]. Lycopene has been also reported to improve the status of enzymatic (superoxide dismutase, catalase, and peroxidase) and nonenzymatic antioxidants (vitamins E and C) in the cell and act as important antioxidant [33].

### 4.2. Anticancer Activities

Carcinogenesis and atherogenesis prevention is possible by lycopene as lycopene protects cellular biomolecules such as lipoproteins, lipids, DNA, and proteins. Diets which are free of tomato or lycopene resulted in increased lipid oxidation in humans. Prostate cancer patients have higher oxidation of proteins and serum lipids levels and lower lycopene levels. Thus, increase in rate of reactive singlet oxygen results due to acyclic molecular structure and apolar characteristics. Lycopene protects humans from prostate and colorectal cancers as suggested by epidemiological studies. In some tissue culture experiments, lycopene has been shown to inhibit cancer cell growth by posing inhibition in the cell cycle and inducing apoptosis in the cancerous cells [10, 34]. Lycopene has been established as an important molecule for inhibition of breast cancer cell proliferation by attenuating the insulin-like growth factor 1 receptor (IGF-1R) pathway [35].

### 4.3. Cardiovascular Effects

Today, the main cause of mortality and morbidity is cardiovascular diseases. There are many identified risk factors for cardiovascular diseases. Proinflammatory status, production of reactive oxygen species, and dysregulated plasma lipid levels cause cardiovascular diseases (CVDs). Most of the risk factors can be modified, and one of the most important ones is diet. Cardiovascular health is firmly associated with a healthy diet. One of the important elements of a cardioprotective diet is that it is rich in fruits and vegetables [36, 37]. The Mediterranean diet supports the consumption of large amounts of vegetables and fruits and is high in tomato consumption. The lower incidence of heart diseases in Mediterranean countries, a healthy diet and good consumption of lycopene, which is known to reduce the risk of CVD, can be shown. Lycopene is also an important cardioprotective with its ability to modulate several important events such as apoptosis and inflammation [3, 38, 39].

CVDs and atherosclerosis have a complex pathophysiology. One of them is oxidation of low-density lipoprotein (LDL). After oxidation, LDL accumulates to a subendothelial matrix and causes formation of foam cells. And also, oxi-LDL causes endothelial dysfunction. In endothelial dysfunction, nitric oxide (NO) proliferation decreases. NO is one of the most important vasodilators which is synthesized by endothelial cells. With NO, blood pressure, which is a risk factor of CVD, decreases. In some studies, lycopene created an improvement in high-density lipoprotein (HDL) functionality [40]. It is a well-known fact that atherosclerosis is an inflammatory process. Lycopene decreases inflammation with effects on neutrophils and macrophages [41]. In fact, lycopene shows cardioprotective effects by affecting many steps of atherosclerosis.

### 4.4. Neurobiological Effects

The neurobiological antioxidant effects of carotenoids and lycopene in particular have attracted clinical attention. This in turn has prompted research with an ever-increasing interest in lycopene's health benefits. Interestingly, lycopene is found to be the leading carotenoid in the plasma of tissues of a US-based population of healthy individuals. In addition to that, lycopene is among the most potent antioxidants which efficiently neutralize the reactive oxygen species. Being a potent antioxidant, the preventive and therapeutic benefits of lycopene have been studied and reported by several literatures [42]. In addition to a wide range of reported health benefits of lycopene, it is known to act as a natural neuroprotective agent. Accordingly, the therapeutic and brain-health benefits of lycopene are worth being further examined in neurological, cognitive, and psychobehavioral disease conditions. In the same vein, some earlier research works have confirmed lycopene's positive effects on neurodegenerative diseases, including Alzheimer's and Parkinson's [43].

In preclinical studies and at system neuroscience and neurobiological levels, the anti-inflammatory properties of lycopene are shown to partly depend upon the oxidative stress, memory retrieval, mitogen-activated protein kinase (MAPK)/extracellular signal-regulated kinase (ERK) pathway, and apoptosis. Mechanistically, lycopene seems to potentially upsurge the protein expression of tyrosine receptor kinase B expression and subsequently its downstream cascades MAPK/ERK1/2/cAMP response element-binding protein (CREB)/brain-derived neurotrophic factor (BDNF) signaling pathways. The antiapoptotic properties of lycopene are associated with preserving the B cell leukemia/lymphoma-2 (Bcl-2) gene expression. This would be concurrently modulated through downscaling the Bcl-2-associated X protein (Bax) gene expression and decreasing the caspase 3 activity [44, 45].

### 4.5. Lycopene and Sleep

The cross-link between sleep quantity/quality and diet has received research focus. Some studies have examined the association between sleep duration and lycopene-rich fruit/vegetable intakes. In such studies, sleep parameters are tracked using self-reports, and diet is assessed by food diaries. To examine the relatable impact of a lycopene-rich diet on sleep, foods containing fruit/vegetable should be disaggregated in subjects' diet records. Based on the multiple regression adjusting for confounders, analyses have shed light on the link between sleep biology parameter dynamics and lycopene-rich diet. Indeed, the association between sleep duration and plasma total carotenoids and lycopene supports the positive impact of a lycopene-rich diet on sleep parameters. Based on known information from previous literature, the importance of lycopene in diet holds research, practice, and policy implications [46].

### 4.6. Anti-Inflammatory Effect

Inflammation is a type of immune response mechanism activated against different harmful stimuli to eliminate them from one side and initiate the healing process from the other side. It is a very important step in tissue regeneration, repairing, and remodeling and even for regulating the tissue hemostasis (in subtle forms). The inflammatory response is accompanied by the activation of some cellular signaling pathways in the injured tissue that attract inflammatory blood cells and also regulate the levels of inflammatory mediators. Despite its brilliant effects, the uncontrolled form of inflammation is accompanied by creating different types of chronic diseases, like cancer, neurodegenerative disease, diabetes, bowel diseases, and arthritis [47, 48].

The promotion of inflammation is regulated by the inflammatory mediators which contain different types of cytokines (like interleukin 1 (IL-1), IL-5, IL-6, interferon-gamma (IFN*γ*), and tumor necrosis factor *α* (TNF-*α*)), chemokines (such as IL-8, vascular cell adhesion molecule 1 (VCAM-1), and monocyte chemoattractant protein- (MCP-) 1), prostaglandins, free radicals, enzymes (like matrix metalloproteinase (MMP) and cyclooxygenase (COX)), and growth factors [49]. The activation of these mediators leads to the increasing of the permeability of the vascular endothelial near the targeted tissue followed by the employment of immune reagents (like neutrophils and complement factors) which eliminate the destructive agent as well as promote the healing [50]. Controlling the inflammation process could be managed via regulating the expression of these mediators. So far, several types of anti-inflammatory agents have been introduced that resulted from both natural and chemical sources including flavonoids, glucocorticoids, angiotensinogen-converting enzyme (ACE) inhibitors, phenolics, terpenoids, carotenoids, retinoids, and different types of nonsteroidal anti-inflammatory drugs (NSAIDs) [51–56].

**Figure 4:**
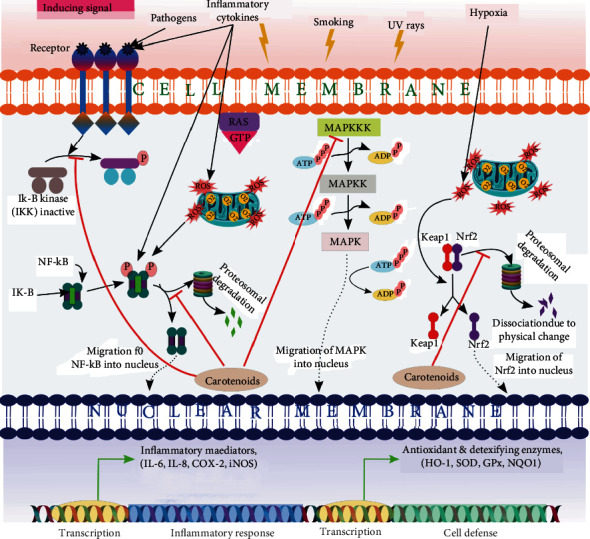
Scheme of anti-inflammatory effect of carotenoids on different inflammatory signaling pathways [60].

As a type of dietary carotenoid (Figure 4), lycopene shows anti-inflammatory effects that are resulted from its lipophilic nature, which has a close association with the cell membrane and enables them to regulate the inflammatory mediator signaling pathways and activating the expression of antioxidant genes. Lycopene could prevent the production of different types of cytokines (like IL1, IL6, IL8, and TNF-*α*), chemokines, nitric oxide (NO), and cyclooxygenase that could modulate the immune system reaction [57]. It could also inhibit the nuclear factor kappa B (NF-*κ*B) signaling pathway (which is known as one of the reasons for inflammation reaction) via attachment to the IkB protein (inhibitor of nuclear factor kappa B) and thus maintain its attachment to NF-*κ*B and prevent its translocation to the nucleus [58]. Accordingly, lycopene exerts its anti-inflammatory effect via two main strategies: (1) preventing the inflammation mediators' positive feedback loop and (2) stimulating the negative feedback mechanism [59].

This anti-inflammatory property of lycopene introduces it as a potent agent for the treatment of cancer, affecting human health, inhibition of metastasis and tumor progression, prevention of metabolic disorders related to obesity, prevention of neurodegenerative diseases (via suppression of neuroinflammatory signaling), etc. [61–64].

It could be applied for the treatment of vascular inflammatory diseases. It has the ability to prevent the proinflammatory responses mediated by the high mobility group box 1 (HMGB1) in HMGB1-activated primary human umbilical vein endothelial cells (HUVECs) via eliminating the expression of HMGB1 receptors, cell adhesion molecules (CAMs), receptors for advanced glycation end products (RAGE), and toll-like receptor (TLR) 2 and 4. These events lead to the inhibition of HMGB1 releasing mediated by lipopolysaccharide (LPS), prevention of the HMGB1-mediated tumor necrosis factor- (TNF-) secretory phospholipase A2 (sPLA2)-IIA expressing, and thus downregulation of the proinflammatory signaling mediated by HMGB1 in endothelial cells [65].

**Figure 5:**
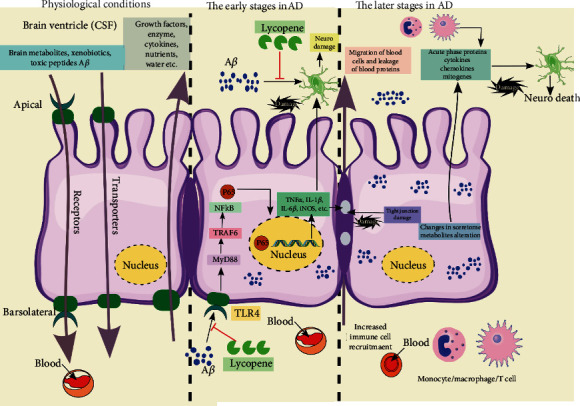
The anti-inflammatory effect of lycopene against *β*-amyloid-mediated inflammation. It shows its anti-inflammatory effect via preventing the activation of TLR4 (and thus the NF-*κ*B) and has direct effect on disaggregation of A*β* [66].

Lycopene showed protective effects in *β*-amyloid-mediated inflammation and could improve the learning and memory functional deficits via reducing the expression of toll-like receptor 4 (TLR4) and NF-*κ*B p65 mRNA and the amount of TNF-*α*, IL-1*β*, and IL-6*β* in serum. These events could eliminate the deposition of *β*-amyloid in the hippocampus tissues (Figure 5) [66].

**Figure 6:**
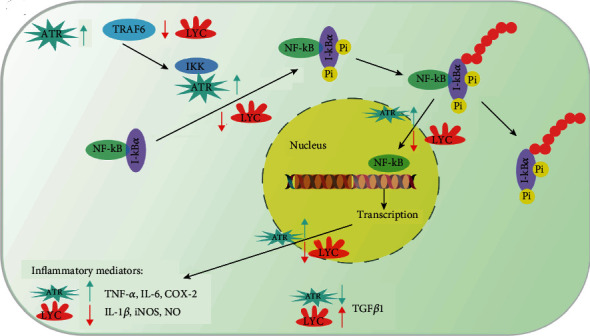
Schematic of lycopene anti-inflammatory effect in ATR-mediated cardiac injury, which is conducted via the TRAF6-NF-jB pathway.

It could also reduce the atrazine- (ATR-) induced cardiac injury by reducing the inflammatory markers. Indeed, it could modulate the amount of NOS (nitric oxide synthase) and NO (nitric oxide) content and reduce the production of cytokines and chemokines via blocking the NF-*κ*B pathway (Figure 6) [67].

### 4.7. Antihypertensive and Antiaggregative Effect

Events mediated by reactive oxygen species contribute severely to cardiovascular remodeling and endothelial dysfunction in hypertension and other CVDs. These contributions are due to effects on apoptosis, endothelial function, hypertrophy, angiogenesis, and sparseness of capillaries and else. Recently, the positive effects of lycopene on cardiovascular health have been indicated by in vitro and in vivo studies. These positive effects are largely mediated by (1) protection against hypertrophy by improving ROS production, (2) myocardial ischemia/reperfusion (I/R)—inhibition of injury caused by endoplasmic reticulum stress, (3) preventing LDL from oxidative damage, (4) improving ventricular remodeling after myocardial infarction, and (5) enhancing endothelial function [68].

In a study on overweight and obese individuals, the lycopene/uric acid ratio and lycopene were associated with a low prevalence of hypertension. In a different study, it has been observed that lycopene may exert antihypertensive effects in normotensive rats without causing hypotension and affect angiotensin-II-induced cardiovascular remodeling [68, 69].

In a study of 54 patients with hypertension, a decrease in blood pressure was reported after 6 weeks of regular tomato extract supplementation. Another study found that lycopene supplementation of more than 12 mg/day in hypertensive patients could significantly reduce diastolic blood pressure. The same effect was not seen in systolic blood pressure. Another important effect of lycopene is its concentration-dependent antiplatelet activity. Platelets play a role in the pathogenesis of atherosclerotic plaque, development of acute thrombotic events, and restenosis after endovascular procedures. While explaining the antiplatelet effect of lycopene, various mechanisms such as the interaction with thromboxane, thrombin, collagen, von Willebrand factor, P-selectin, and inflammatory mediators and the influence on calcium and cyclic guanosine monophosphate signaling and ADP-mediated aggregation were taken into account [70].

## 5. Conclusion

This review article provides an update on the sources as well as biological and pharmacological profile of lycopene, a pharmacologically active carotenoid, a major bioactive constituent in many plants mainly from tomato that possesses many pharmacological benefits such as anticancer, antioxidant, cardioprotective, and antihypertensive effects. The antioxidant potential of tomatoes and its products is mainly attributed to the lycopene that also holds an imperative role in maintaining human health. The proven positive effect of lycopene on cardiovascular diseases is known. The reasons for this effect can be attributed to the positive effects of lycopene on serum lipid levels, their reduction of endothelial dysfunction, positive effects on inflammation, their help in blood pressure regulation, and antioxidative effects.

It is evident from the data presented in the manuscript that there are sufficient in vitro studies which have been investigated based on lycopene, but there is further need for in vivo and clinical investigations. The safety aspects of lycopene need further in-depth studies. Also, the exact molecular mechanism of various biological activities of lycopene needs further research.

## Data Availability

The data supporting this review are from previously reported studies and datasets, which have been cited. The processed data are available from the corresponding author upon request.
